# *Cissampelos sympodialis* Eichl. (Menispermaceae), a medicinal plant, presents antimotility and antidiarrheal activity *in vivo*

**DOI:** 10.1186/s12906-015-0578-7

**Published:** 2015-07-25

**Authors:** Igor Rafael Praxedes de Sales, Flavia Danniele Frota Machado, Alexsandro Fernandes Marinho, Ana Sílvia Suassuna Carneiro Lúcio, José Maria Barbosa Filho, Leônia Maria Batista

**Affiliations:** Programa de Pós-Graduação em Produtos Naturais e Sintéticos Bioativos, Centro de Ciências da Saúde, Universidade Federal da Paraíba, Campus de João Pessoa, 58051-900 Cidade Universitária João Pessoa-PB, Brazil; Departamento de Ciências Farmacêuticas, Centro de Ciências da Saúde, Universidade Federal da Paraíba, Campus de João Pessoa, 58051-900 Cidade Universitária João Pessoa-PB, Brazil

**Keywords:** Gastrointestinal motility, Diarrhea, Cissampelos sympodialis

## Abstract

**Background:**

*Cissampelos sympodialis* (Menispermaceae), known as “Milona” has a specific verified medicinal use for the treatment of diarrhea and respiratory tract diseases. This work aims to evaluate the antimotility and antidiarrheal activity of crude ethanolic extract (EtOHE-*Cs*), and the total alkaloid fraction (TAF-*Cs*) obtained from aerial parts of *C. sympodialis*.

**Methods:**

Normal intestinal transit and gastric emptying were used to evaluate antimotility activity. Castor oil-induced diarrhea and castor oil-induced enteropooling were used to evaluate antidiarrheal activity.

**Results:**

The results indicated that EtOHE-*Cs* has no antimotility activity, but did demonstrate antidiarrheal activity (at 500 mg/kg), and this activity is related to reduction of intestinal fluid accumulation. The TAF-*Cs* (at 250 and 500 mg/kg) showed antidiarrheal activity by reducing gastrointestinal motility (gastric emptying and normal intestinal transit).

**Conclusions:**

The antidiarrheal activity of *C. sympodialis* can be attributed to the chemical compounds already isolated and quantified in this species, mainly alkaloids.

## Background

Diarrhea is characterized by an increase in the frequency of bowel movements, abdominal pain, and bowel discharge of semisolid or watery fecal matter (three or more times in a day) [1,2]. Control of gastrointestinal motility is very complex involving multiple signals, such as nitric oxide (NO), gastrin, ghrelin, prostaglandins, 5-hydroxytryptamine (5-HT), dopamine, catecholamine, and acetylcholine [3,4]. The main causes of diarrhea are imbalances in the pathways just mentioned, as well as infectious agents, plant toxins, and inflammatory problems [5]. Worldwide, the disease affects around 2.2 million people annually, and those most affected are children under the age of five years [6]. Changes in gastric emptying coordination cause not only poor nutrient digestion and absorption, but also the development of diseases. Fully 25-40% of patients with functional dyspepsia report delays in gastric emptying. The development of duodenal ulcers is often related to the stimulation of gastric emptying, since acid content is not completely neutralized in the duodenum [7-10].

Medicinal herbs constitute the majority component of traditional medicines as they are practiced worldwide; this is due to their economic viability, accessibility, and ancestral experiences [11]. Thus, for gastrointestinal disorders, (such as diarrhea) researching medicinal herbs has become very important in developing new therapies.

*Cissampelos sympodialis* Eichl, belongs to the family Menispermaceae, and is known as “bindweed”, and in Brazil as “milona”, “jarrinha”, or “orelha-de-onça”. It is widely used by Indian tribes, and in folk medicine to treat various diseases such as diarrhea, diseases of the genitourinary tract, and especially respiratory tract diseases such as asthma [12]. The species was selected for study because of ethno-pharmacological and chemotaxonomic criterion.

Several chemical compounds belonging to the alkaloids class have been isolated from the specie’s leaves and roots, as examples the: bisbenzylisoquinolinic (warifteine, methylwarifteine, roraimine, and simpodialine); morphinic (milonine); aporphinic (laurifolin) and oxo-aporphinic (liriodenine) alkaloids [13,14]. Quality control studies have shown that both alcoholic fractions of the leaves (AFLs), and alcoholic fractions of the roots (AFRs) present alkaloids as their principal compounds, being warifteine the chemical marker of both AFL and AFR [15]. Crude ethanolic extract (EtOHE-*Cs*) was standardized using warifteine and methylwarifteine as markers that were also found in the total alkaloid fraction (TAF-*Cs*) [16]. The aqueous fraction of the EtOHE-*Cs* has shown spasmolytic activity on tracheal smooth muscle, and this activity involving inhibition of phosphodiesterase (PDE) and increased levels of cyclic adenosine monophosphate (cAMP) in guinea pig trachea. Warifteine, a bisbenzylisoquinolinic alkaloid obtained from *C. sympodialis* also has shown spasmolytic activity by inhibition of calcium channels (in the rabbit thoracic aorta) and activation of potassium channels (in the rat thoracic aorta) [13, 17, 18]. Thus, considering the studies cited above and other studies reporting the antidiarrheal activity of alkaloids [19] probably alkaloids of *C. sympodialis* can be used in diarrhea therapy. Based on its popular use and its spasmolytic activity “in vitro”, the aim of this study was to evaluate the antimotility and antidiarrheal activities of EtOHE-*Cs* and TAF-*Cs* “in vivo” obtained from aerial parts of *C. sympodialis.*

## Methods

### Materials

Metoclopramide hydrochloride 10 mg (SANOFI-AVENTIS®; Brasil); loperamide hydrochloride 2 mg (JANSSEN-CILAG®; Brasil); phenol red (VETEC®; Brasil), charcoal meal (VETEC®; Brasil); and Tween 80 (MERCK®; Germany).

### Plant material and extraction

Aerial parts of *C. sympodialis* were collected from the garden of the “*Centro de Biotecnologia*” (CBIOTec/UFPB) in March 2013, and identified by Dr. Maria de Fátima Agra (*Laboratório de Farmacobotânica* - CBIOTec/UFPB). A voucher specimen was deposited in the “*Herbário Lauro Pires Xavier*” Herbarium, No. 1456. To obtain the crude ethanolic extract, dried and pulverized material from aerial parts of *C. sympodialis* (4000 g) were subjected to maceration in 95% ethanol for 72 hours. After extraction, the extractive solution was concentrated in a rotary evaporator under reduced pressure at a temperature of 45°C, yielding 300 g of crude ethanolic extract (EtOHE-*Cs*). A 100 g aliquot of the EtOHE-*Cs* was solubilized in an acid solution (3% HCl), and filtered with filter paper. The resulting acid solution was subjected to liquid-liquid partition with dichloromethane. The dichloromethane layer was discarded, and the acid layer was basified with NH_4_OH to pH = 9, with a subsequent extraction into chloroform. The chloroform phase was filtered, anhydrous sodium sulfate was added, and the liquid was concentrated in a rotary evaporator resulting in the TAF-*Cs* (4.8 g).

### Experimental animals

Swiss adult male mice (*Mus musculus)* weighing between 25-35 g were used for the experiments. The animals from the “*Biotério Professor Thomas George*” (UFPB) were kept at temperatures between 23-25°C, with a 12-hour light/dark cycle in the animal house. The animals were fed Labina, and water *ad libitum*. For the experiments, they were randomly distributed into different experimental groups. All experiments were started in the morning, and the experimental procedures were approved by the “*Comitê de Ética em Pesquisa Animal*” (CEPA/CBIOTec/UFPB), and recorded as No. 0705/06, in accordance with international principles for research with laboratory animals [20].

### Effect of *C. sympodialis* on gastric emptying

Adult mice, (fasted for 12 h) were randomly divided into seven groups (n = 7). Mice in the first group received 12% Tween 80 solution - vehicle (10 mL/kg), the second group received metoclopramide (30 mg/kg), and the other groups received 62.5, 125, 250, and 500 mg/kg of EtOHE-*Cs,* or TAF-*Cs*, the seventh group (zero time control) received saline solution 0.9%, all by via oral (v.o.). After 1 h of administration (extract, fraction and drugs), a suspension of phenol red marker (0.05%) in carboxymethylcellulose (1.5%) (10 mL/kg) was also given to each animal (v.o.). The zero time control group was euthanized (by cervical dislocation) immediately after the administration of the marker, and the other groups, after 30 min. The abdominal cavity was opened, the pylorus and the distal portion of the esophagus were clipped, the stomach was removed and opened, and its contents were washed with 7 mL of distilled water. The gastric contents collected were centrifuged at 450 g for 15 min, and 1 mL from the supernatant was mixed with 1 mL of 0.025 M NaOH (pH = 12). Afterwards, 150 μL of the homogenate was pipetted in duplicate in a 96 well plate, and a spectrophotometric reading was made using a 560 nm filter. The results were expressed as the percentage of gastric emptying compared to the zero time control using the formula below [21].

$$ \%\  gastric\  emptying = 100\ \hbox{--} \frac{mean\  absorbance\  of\  sample\ x\ 100}{mean\  absorbance\  of\  the\  zero\  time\  control\  group} $$

### Effect of *C. sympodialis* on normal intestinal transit

The method previously described by Stickney and Northup [22] was used with modifications. Adult mice were fasted for 24 hours, and were randomly divided into six groups (n = 7). Mice in the first group received 12% Tween 80 solution - vehicle (10 mL/kg); the second group received metoclopramide (30 mg/kg) or loperamide (5 mg/kg); and the other groups received 62.5, 125, 250, and 500 mg/kg of EtOHE-*Cs,* or TAF-*Cs*, respectively, all by the oral pathway (v.o.). At 1 hour from these administrations, a suspension of charcoal meal (0.5%) in carboxymethylcellulose (0.5%) (10 mL/kg) was given to each animal (v.o.). After 30 minutes, the animals were euthanized by cervical dislocation to remove the small intestine. The transit percentage was calculated on the basis of distance traveled by the charcoal meal as divided by the total length of the intestine, using the formula below.

$$ \%\  transit = \frac{distance\  traveled\ by\  charcoal\  meal}{\  total\  length\  of\  the\  intestine}x\ 100 $$

### Effect of *C. sympodialis* on castor oil-induced diarrhea

For the evaluation of antidiarrheal activity was used as a basis the methodology described by Awouters and collaborators [23] with some local modifications. Adult mice were fasted for 12 h and were randomly divided into six groups (n = 5–8). Mice in the first group received 12% Tween 80 solution - vehicle (10 mL/kg), the second group received loperamide (5 mg/kg), and the other groups received 62.5, 125, 250, and 500 mg/kg of EtOHE-*Cs,* or TAF-*Cs*, respectively, all via oral (v.o.). At 1 hour from these administrations, castor oil was given to each animal (v.o.) 10 mL/kg. Following the administration of castor oil, the animals were placed in separates cages containing transparent blotting papers for observation of the total number of feces, and their quantification (liquid, semi-solid, and solid) during 4 h. After this, all the animals were euthanized by cervical dislocation. The following parameters were monitored: evacuation classification – 1 (normal stool), 2 (semi-solid stool), and 3 (watery stool) and evacuation index (EI), expressed according to the formula:

$$ EI=1\times \left( no.\  of\  type\ 1\  stools\right) + 2\times \left( no.\  of\  type\ 2\  stools\right) + 3\times \left( no.\  of\  type\ 3\  stools\right) $$

### Effect of *C. sympodialis* on castor oil-induced enteropooling

Animals (adult male mice) were fasted for 12 hours and were randomly divided into three groups (n = 7). Mice in the first group received 12% Tween solution 80 - vehicle (10 mL/kg), the second group received loperamide (5 mg/kg), and the third group received 500 mg/kg of EtOHE-*Cs*, (the only dose that showed antidiarrheal activity), all by oral pathway (v.o.). At 1 h from the administrations, the animals were euthanized by cervical dislocation, laparotimized, and then the pyloric and caecal ends of the small intestine were tied and the intestines were removed. The content of each intestine was measured in a graduated measuring cylinder, and the volume was noted according to Ezeja and Anaga [24]_._

### Statistical analysis

The parametric data were expressed as the mean ± standard deviation (SD) and non-parametric data were expressed as median (minimum value – maximum value). This data was subjected to variance analysis (ANOVA), followed by a Dunnett’s test (parametric) or Kruskal-Wallis test followed by Dunn’s multiple comparison test (non-parametric). The minimum level of significance was p < 0.05 in all analyses. For the data processing, INSAT (GraphPad Software^©^ Inc., San Diego, CA, USA) software was used.

## Results and discussion

### Effect of *C. sympodialis* on gastrointestinal motility: normal intestinal transit and gastric emptying

**Figure 1:**
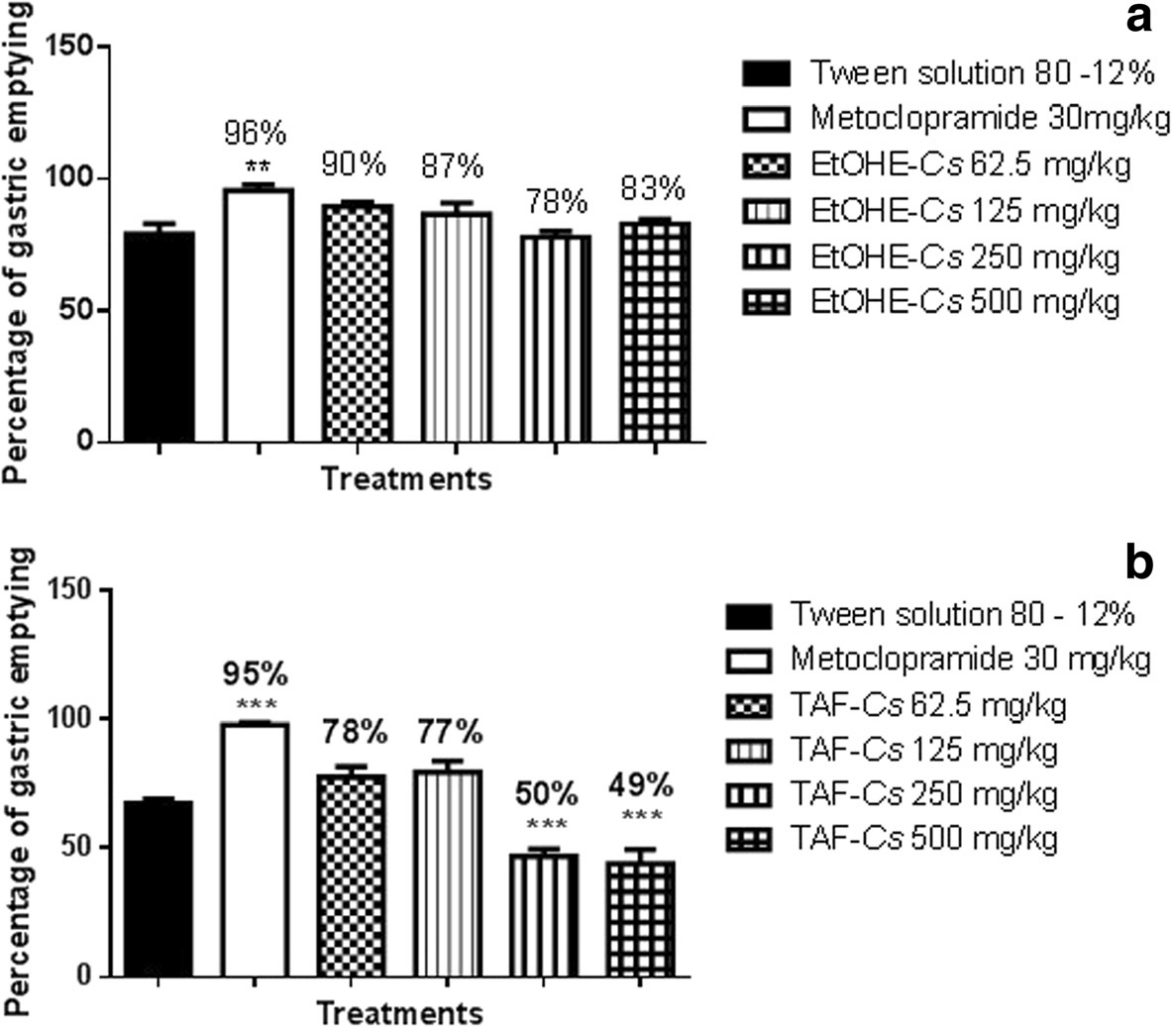
**Effect of oral administration of EtOHE-Cs (a) and TAF-Cs (b)** **on gastric emptying in mice.** **(a)** ANOVA: F_(5,27)_ = 6.803 (p <0.05) (n = 5–7) followed by Dunnett’s test (** p <0.01 compared to the 12% Tween solution group). **(b)** ANOVA: F_(5,29)_ = 40.29 (p <0.05) (n = 5–7) followed by Dunnett’s test (*** p <0.001 compared to the 12% Tween solution group).

**Fig. 2:**
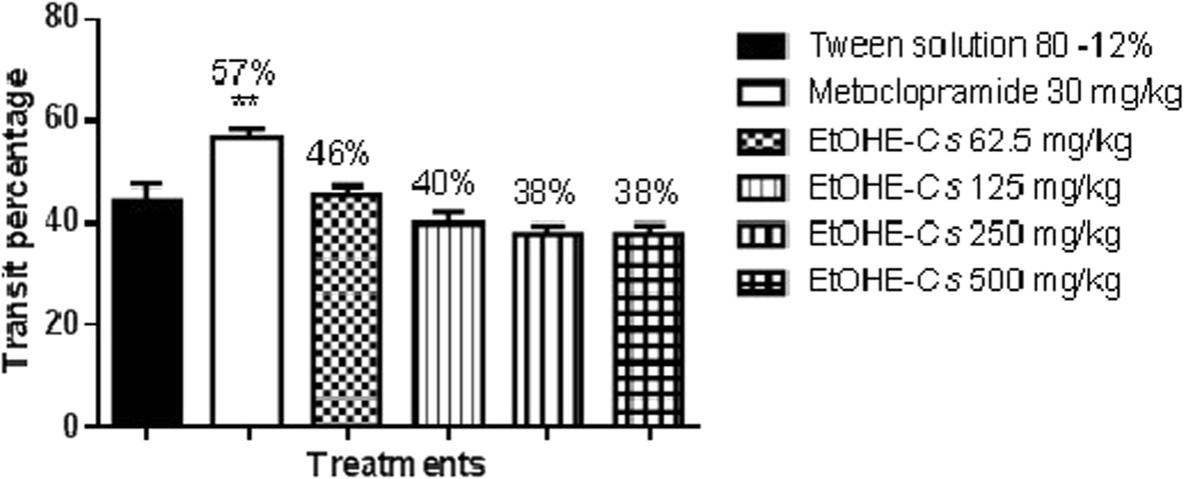
**Effect of oral administration of EtOHE-Cs** **on normal intestinal transit in mice.** ANOVA: F_(5,31)_ = 9.47 (p <0.05) (n = 5–7) followed by Dunnett’s test (** p <0.01 compared to the 12% Tween solution group).

**Figure 3:**
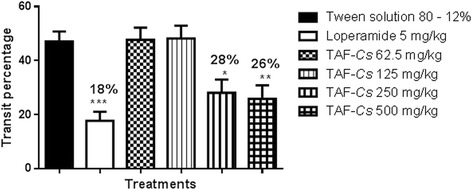
**Effect of oral administration of TAF-Cs on normal intestinal transit in mice.** ANOVA: F_(5,32)_ = 9.175 (p <0.05) (n = 6–7) followed by Dunnett’s test (* p < 0.05 ** p <0.01 *** p < 0.001 compared to the 12% Tween solution group).

The antimotility activity of EtOHE-*Cs* and TAF-*Cs* was investigated by measuring gastric emptying, and normal intestinal transit in mice. Earlier studies have reported that antimotility and antidiarrheal properties of medicinal plants are due to tannins, alkaloids, saponins, and sterols [25]. Normal intestinal transit and gastric emptying were not altered for any dose (62.5, 125, 250 and 500 mg/kg) evaluated for EtOHE-*Cs,* however TAF-*Cs* (at 250 and 500 mg/kg) reduced gastric emptying and normal intestinal transit, as expressed in Figures 1a, 1b, 2 and 3. The results suggest that *C. sympodialis* alkaloids affect gastrointestinal motility, and are likely to present spasmolytic activity “in vivo”. Though showing antimotility activity, we commenced investigations of anti-diarrheal activity, because diarrhea is not only caused by motility deregulation. It is also caused by microorganism infections, hyper secretion of intestinal fluids, and inflammatory bowel disease [26].

### Effect of *C. sympodialis* on castor oil-induced diarrhea and enteropooling

Diarrhea is a disease that has many clinical signs such as hyper-propulsive motility of gastrointestinal tract, and hyper-secretion throughout the intestinal mucosa. Animal models are commonly used to induce experimental diarrhea [26, 27], and to study a plants’ mechanisms of action and active principles.

Castor oil, prostaglandin E_2_ (PGE_2_), and heat-labile enterotoxin are commonly used to induce diarrhea in animals. The induction of diarrhea by castor oil is recommended for study of the anti-secretory and antimotility potential of medicinal plants. “Castor oil diarrhea” is due to its active metabolite, ricinoleic acid that is released by the action of intestinal lipases [28].

The liberation of ricinoleic acid results in irritation and inflammation of the intestinal mucosa, and is associated with the release of nitric oxide, prostaglandin, and other autacoids [28, 29]. The enteropooling induced by castor oil, probably occurs through stimulation of cyclic AMP/GMP production, and phosphorylation of cystic fibrosis transmembrane conductance regulators (CFTRs). This consequently leads to intestinal motility stimulation and to increased secretion of fluids and electrolytes (mainly Cl^−^ and Na^+^) [30,31].

In castor oil induced-diarrheic animals, the EtOHE-*Cs* in its highest dose (500 mg/kg) significantly reduced the evacuation index as can be seen in Table 1, and TAF-*Cs* showed similar antidiarrheal effects at doses of 250 and 500 mg/kg, as can be seen in Table 2.

**Table 1:** **Effect of oral administration of EtOHE-Cs, and loperamide on castor oil induced diarrhea in mice**

**Treatment (v.o.)**	**Dose (mg/kg)**	**Evacuation Index**
		**(EI)**
**12** **%** **Tween 80 solution**	**-**	**13.0 (12.0 - 18.0)**
**Loperamide**	**5**	0.0 (0.0 - 1.0)**
EtOHE-*Cs*	**62.5**	**12.0 (7.0 - 14.0)**
EtOHE-*Cs*	**125**	**13 (9.0 - 19.0)**
EtOHE-*Cs*	**250**	**11.5 (8.0 - 17.0)**
EtOHE-*Cs*	**500**	4.0 (2.0 - 5.0)*

Data are presented as median (minimum value – maximum value). Kruskal-Wallis test followed by Dunn’s multiple comparison test (* p <0.05, ** p <0.005 compared to the 12% Tween solution group). The EtOHE-Cs reduced evacuation index (500 mg/kg) compared to control group (12% Tween 80 solution).

**Table 2:** **Effect of oral administration of TAF-** ***Cs,*** **and loperamide on castor oil induced diarrhea in mice**

**Treatment (v.o.)**	**Dose (mg/kg)**	**Evacuation Index (EI)**
**12** **%** **Tween 80 solution**	**-**	**15.0 (10.0 - 23.0)**
**Loperamide**	**5**	0.0 (0.9 - 2.0)***
TAF-*Cs*	**62.5**	**13.5 (6.0 - 16.0)**
TAF-*Cs*	**125**	**6.0 (4.0 - 8.0)**
TAF-*Cs*	**250**	1.0 (0.0 - 5.0)**
TAF-*Cs*	**500**	1.0 (0.0 - 2.0)**

Data are presented as median (minimum value – maximum value). Kruskal-Wallis test followed by Dunn’s multiple comparison test (** p <0.05, *** p <0.001 compared to the 12% Tween solution group). The TAF-Cs reduced evacuation index (250 and 500 mg/kg) compared to control group (12% Tween 80 solution).

**Figure 4:**
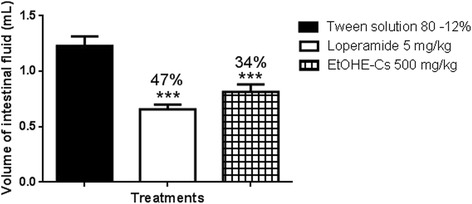
**Effect of oral administration of EtOHE-Cs on castor oil induced enteropooling in mice.** ANOVA: F_(2,18)_ = 18.93 (p <0.05) (n = 7) followed by Dunnett’s test (*** p <0.001 compared to the 12% Tween solution group).

The EtOHE-*Cs* does not have antimotility activity. Due to this, in another step we investigated if the extract had intestinal anti-secretory activity. For the enteropooling study, the EtOHE-*Cs* (at 500 mg/kg) significantly reduced the intraluminal fluid volumes of the intestinal contents (Figure 4), suggesting that the EtOHE-*Cs* antidiarrheal activity involves the reabsorption of water and electrolytes (such as Na^+^), and probably, the involvement of prostaglandins [28].

## Conclusions

The antidiarrheal activity of *C. sympodialis* can be attributed to chemical compounds already isolated and quantified in this species, such as flavonoids and principally the alkaloids warifteine and methylwarifteine [32]. These compounds are known to inhibit autacoid and prostaglandin release [33]. Inhibition of prostaglandin E_2_ (PGE_2_) is known to reduce secretory response in the intestine, and to inhibit gastrointestinal motility [34].
